# Hepatitis E Virus Infection in Central China Reveals No Evidence of Cross-Species Transmission between Human and Swine in This Area

**DOI:** 10.1371/journal.pone.0008156

**Published:** 2009-12-07

**Authors:** Wen Zhang, Shixing Yang, Liping Ren, Quan Shen, Li Cui, Kezhang Fan, Fen Huang, Yanjun Kang, Tongling Shan, Jianzhong Wei, Haifeng Xiu, Yifang Lou, Junfeng Liu, Zhibiao Yang, Jianguo Zhu, Xiuguo Hua

**Affiliations:** 1 School of Agriculture and Biology, Shanghai JiaoTong University, Shanghai, People's Republic of China; 2 School of Medical Science and Laboratory Medicine, Jiangsu University, Zhenjiang, Jiangsu, People's Republic of China; 3 School of Life Science, Fuyang Normal University, Fuyang, People's Republic of China; 4 College of Animal Science and Technology, Anhui Agricultural University, Hefei, People's Republic of China; 5 Fuyang People's Hospital, Fuyang, People's Republic of China; 6 First People's Hospital of Sixian, Sixian, People's Republic of China; National Institute for Communicable Diseases, South Africa

## Abstract

Hepatitis E virus (HEV) is a zoonotic pathogen of which several species of animal were reported as reservoirs. Swine stands out as the major reservoir for HEV infection in humans, as suggested by the close genetic relationship of swine and human virus. Since 2000, Genotype 4 HEV has become the dominant cause of hepatitis E disease in China. Recent reports showed that genotype 4 HEV is freely transmitted between humans and swine in eastern and southern China. However, the infection status of HEV in human and swine populations in central China is still unclear. This study was conducted in a rural area of central China, where there are many commercial swine farms. A total of 1476 serum and 554 fecal specimens were collected from the general human and swine populations in this area, respectively. The seroepidemiological study was conducted by enzyme-linked immunosorbent assay. Conserved genomic sequences of open reading frame 2 were detected using reverse transcription-PCR. The results indicated that the overall viral burden of the general human subjects was 0.95% (14/1476), while 7.0% (39/554) of the swine excreted HEV in stool. The positive rate of anti-HEV IgG and IgM in the serum samples was 7.9% (117/1476) and 1.6% (24/1476), respectively. Phylogenetic analysis based on the 150 nt partial sequence of the capsid protein gene showed that the 53 swine and human HEV isolates in the current study all belonged to genotype 4, clustering into three major groups. However, the HEV isolates prevalent in the human and swine populations were classified into known distinct subgenotypes, which suggested that no cross-species transmission between swine and humans had taken place in this area. This result was confirmed by cloning and phylogenetic analysis of the complete capsid protein gene sequence of three representative HEV strains in the three major groups. The cross reactivity between anti-HEV IgG from human sera and the two representative strains from swine in central China was confirmed by Dot-blot assay. In conclusion, although all the HEV strains prevalent in central China belonged to genotype 4, there is no evidence of cross-species transmission between human and swine in this area.

## Introduction

Hepatitis E virus (HEV), a member of the genus Hepevirus, is a non-enveloped virus with a positive-stranded RNA genome approximately 7.2 kb in length [1]. HEV has been the cause of waterborne outbreaks of hepatitis in Asia and Africa and is a major cause of sporadic hepatitis in these regions. Acute infection primarily affects young adults and is generally self-limiting and resolves in 1–6 weeks, except in women during late pregnancy, among whom 20% mortality has been reported. Chronic HEV infection has recently been reported in transplant recipients [2]. Person-to-person transmission is uncommon [3]. Parenteral and vertical transmission of HEV has been reported, though the role of such transmission in the spread of disease is likely to be limited.

HEV and antibodies to HEV have been found in a wide variety of animals [4]–[7]. It has been hypothesised that zoonosis is involved in the transmission of HEV, especially in non-endemic areas. Swine stands out as a reservoir for hepatitis E virus (HEV) infection in humans,because of the close genetic relationship between swine and human virus and reports of cross-species infection of HEV [8], [9]. The first animal strain of HEV, designated swine HEV, to be isolated and characterized was obtained from a pig in the United States [8]. Subsequently, many HEV samples from swine in over a dozen countries have been identified. Some isolates of swine HEV have been shown to be capable of infecting rhesus monkeys and chimpanzees, although other isolates can only infect swine [10].

HEV isolates were divided into four distinct genotypes according to sequence and phylogenetic analyses. Genotype 1 was previously believed to only infect humans, but this has been disputed recently by a report of this subtype in a pig from Cambodia [11]. Genotype 2 has only been identified in humans in Mexico and Africa (Nigeria, Chad). Genotype 3 is prevalent in swine herds and humans all over the world. Genotype 4 HEV was first detected in humans in 1993 [12] and is mainly distributed in China, Japan, India, Indonesia, and Vietnam. Genotype 4 HEV has a wide host range, being prevalent in humans, swine, and some other animals. Hepatitis E was first recognized in China after a large epidemic in the southern part of the Xinjiang Uighur autonomous region [13]. Since 2000, Genotype 4 HEV has become the dominant cause of hepatitis E disease in China [14]. Recent reports showed that genotype 4 HEV is transmitted between humans and swine in eastern and southern China [9], [15]. The aim of the present study, which was conducted in the swine-farming districts of central China, was to elucidate the relationship between concurrent isolates from swine and humans and thereby ascertain whether swine is a significant source of HEV for human hepatitis E disease in this area.

## Results

### Occurrence of HEV

Table 1 presents the prevalence of HEV antibodies and RNA in 1476 serum samples collected from the general human populations in the central China. The 122 serum samples which were positive for HEV antibodies (IgG or IgM) were analysed further for the presence or absence of HEV RNA by RT-PCR. The results showed that 24 individuals (1.63%) had current infection (i.e., they were reactive for anti-HEV IgM), and 58.3% (14/24) of these infections were accompanied by viremia (i.e., they were positive for viral RNA). The overall viral burden of the study subjects was estimated to be 0.95% (14/1476). This probably approximates the viral burden of the general human population in central China. None of the 98 serum samples which were only positive for IgG tested positive for HEV RNA.

**Table 1:** Prevalence of HEV antibodies and RNA in the general human populations in central China from 2007–2008.

Type of antibody	Positive samples/total analyzed of HEV antibodies [%,(95% CI)]	Positive samples/total analyzed of RNA [%,(95% CI)]
IgG^+^/IgM^−^	98/1476 [6.64, (5.3,7.9)]	0/98 [0]
IgM^+^	24/1476 [1.63, ( 0.9, 2.3)]	14/24 [58.3, ( 36.5, 80.1)]
IgM^+^/IgG^+^	19/1476 [1.29, ( 0.7, 1.9)]	9/19 [47.4, ( 22.3, 72.5)]
IgM^+^/IgG^−^	5/1476 [0.34, ( 0, 0.7 )]	5/5 [100]
Total	122/1476 [8.3, ( 6.8, 9.7)]	14/1476 [0.95, ( 0.4, 1.5)]

Table 2 shows the age-specific prevalence of anti-HEV among the general population in central China from 2007 to 2008. The 40–59 years age group shows the highest prevalence rate of anti-HEV IgG (10.2%), while the 2–19 years age group shows the lowest prevalence rate of anti-HEV IgG (5.1%). No significant difference of anti-HEV IgG prevalence was found between the males and females: 8.1% (63/779) of male and 7.8% (54/697) of females (*P* = 0.462). Our questionnaire showed that 176 individuals had a history of frequent travel to other regions, 9.1% (16/176) of them tested positive for IgG, which was not significantly different from those who did not report frequent travel to other regions (7.8% or 101/1300 *P* = 0.271). Of the 98 individuals who were positive for anti-HEV IgG, only two have ever been admitted to a hospital because of hepatitis E.

**Table 2:** The relation between age and the prevalence of HEV-specific antibodies.

Age	No. of participants	No. (%) of IgG positive	95% CI	No. (%) of IgM positive	95% CI
2–19	297	15 (5.1)	2.4,7.7	3 (1.0)	0,2.3
20–39	376	32 (8.5)	5.6,11.0	10 (2.7)	0.9,4.4
40–59	372	38 (10.2)	7.0,13.4	7 (1.9)	0.4,3.4
≥60	431	32 (7.4)	4.8,10.0	4 (0.9)	0,1.9
Total	1476	117 (7.9)	6.5,9.3	24 (1.6)	0.9,2.3

Table 3 presents the prevalence of HEV RNA in the swine group in central China. The pigs at age of 10–15 weeks old showed the highest prevalence rate of 9.8% (14/143), and the pigs older than 20 weeks showed the lowest prevalence rate 4.6% (5/109). The overall prevalence rate was 7.0% (39/554), and this probably approximates the HEV burden of pigs on swine farms in central China.

**Table 3:** The relation between swine ages and the prevalence of HEV-RNA.

Swine age	No. of analyzed samples	No. of Positive samples (%)	95% CI
<10 weeks	167	11 (6.6)	2.5,10.6
10–15 weeks	143	14 (9.8)	4.6,15
16–20 weeks	135	9 (6.7)	2.1,11.2
>20 weeks	109	5 (4.6)	0.2,9
Total	554	39 (7.0)	4.8,9.3

### Sequence and Phylogenetic Analysis

**Figure 1:**
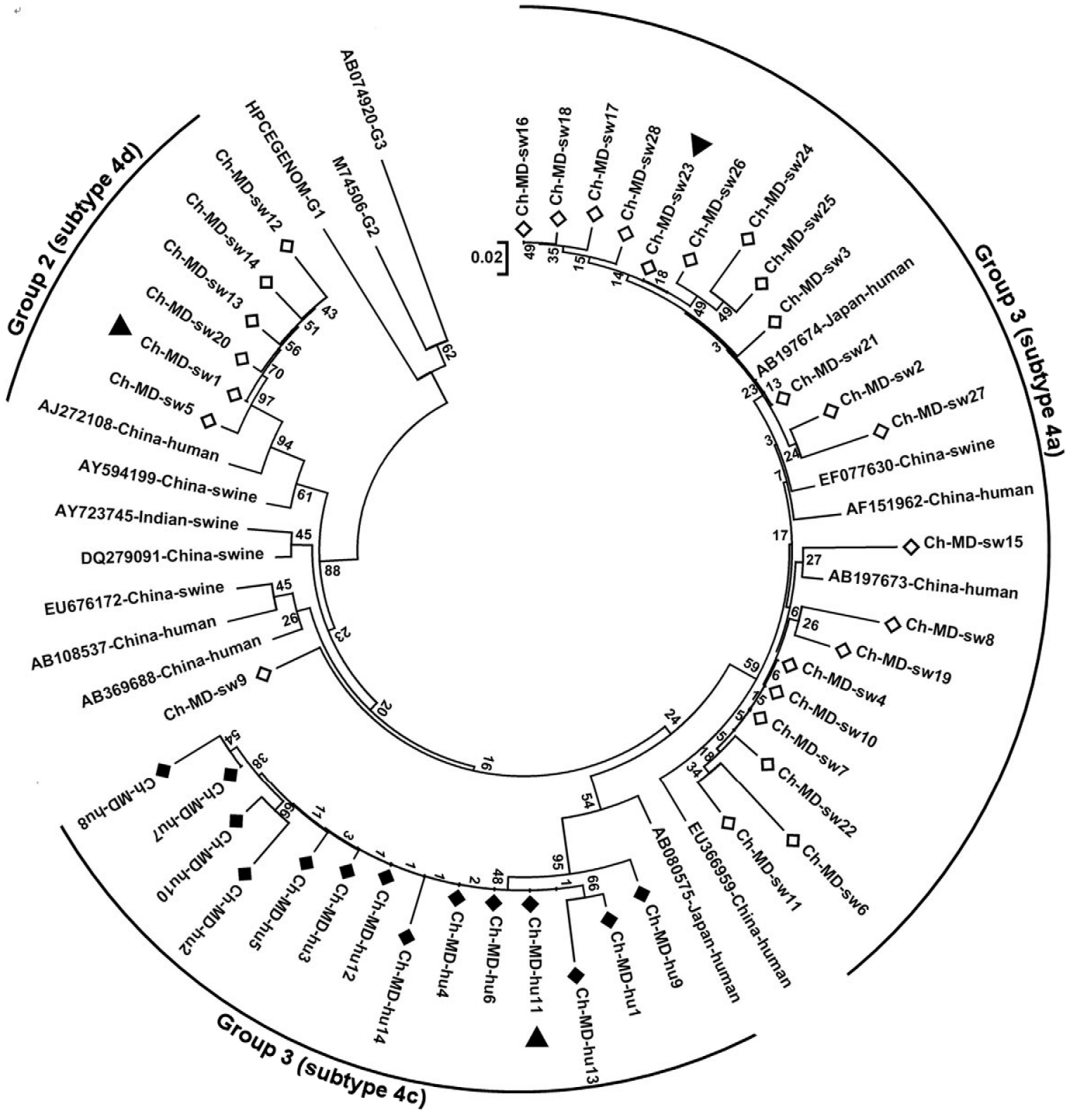
The phylogenetic tree of human (black squares) and swine (white squares) hepatitis E virus (HEV) isolates in central China, 2007–2008. The phylogenetic tree was produced with a 150-nt ORF2 sequence alignments of 42 isolates in the present study and other 16 representative reference sequences. The scale bar indicates a genetic distance of 0.02% nucleotide substitution per position. Values for various branches are percentages of the tree obtained from 1000 resamplings of the data. Genotype 1, 2 and 3 strains are included as outgroup. The three black triangles show the three representative strains whose complete sequences of ORF2 were determined in the present study.

The 150-nt PCR-amplified products from 39 swine and 14 human HEV isolates were sequenced. The results identified 28 swine and 14 human HEV isolates with distinct nucleotide sequences. The sequences were deposited in GenBank, isolate names were from Ch-MD-hu1 to Ch-MD-hu14 and from Ch-MD-sw1 to Ch-MD-sw28, and the corresponding accession numbers were FJ641988-FJ641993 and FJ641994-FJ642021, respectively. A BLAST search using these 42 sequences showed that these isolates all belonged to genotype 4 HEV, and some of the matching sequences from GenBank are included in Figure 1. In order to further classify the 42 genotype 4 HEV strains, phylogenetic analysis was performed including 16 well characterized reference sequences [16]. Results indicated that 41 of the strains determined in the present study partitioned into three main groups, designated group 1, 2 and 3, while the other specimen clustered alone (Fig. 1). Group 1, which consisted of 21 diverse swine specimens, clustered with a bootstrap value of 59%, and had an intra-group diversity which ranged from 0.6% to 7.1%. Reference sequence (AF151962) identified group 1 to be of subtype 4a. Group 2 consisted of 6 swine specimens which group together with a bootstrap value of 97%. Group 2 shows an intra-group diversity from 0.3% to 2.4% and could be classified into subtype 4d according to the reference sequences (AJ272108 and AY594199). Group 3 was also diverse and included all 14 human specimens which group together with a bootstrap value of 97% and have an intra-group diversity ranging from 0.4% to 6.4%. The reference used (AB080575) defined group 3 as subtype 4c. The left four columns of table 4 show the sequence differences between the three groups and the strains with full genome available in GenBank over the 150nt region. From these differences we could conclude that group1, 2 and 3 were most similar to genotype4a, genotype4d and genotype4c, respectively, which confirmed the results of the phylogenetic analysis (Fig. 1).

**Table 4:** The sequence differences between the strains in the present study and the strains with full genome available in GenBank over the 150nt region (for group1–3) or the entire ORF2 (for Ch-MD-sw23, Ch-MD-sw23, and Ch-MD-hu1).

(sub)genotype	Group1	Group2	Group3	Ch-MD-sw23	Ch-MD-sw1	Ch-MD-hu1
Ch-MD-sw1	NA	NA	NA	8.3		
Ch-MD-hu1	NA	NA	NA	10.3	8.3	
Group2	9.9–12.5			NA	NA	NA
Group3	9.4–11.6	10.9–14.8		NA	NA	NA
Genotype1	14.9–17.3	15.4–19.3	15.4–18.8	18.1–20.3	19.8–20.5	19.1–20.3
Genotype2	15.1–18.9	17.7–18.4	15.0–17.7	19.3	19.7	19.9
Genotype3	14.3–23.9	14.7–20.5	14.8–23.9	17.0–18.3	17.3–18.5	17.6–18.9
Genotype4a	***4.0–6.9***	9.5–12.3	9.6–12.5	***7.3***	9.4	10.7
Genotype4c	7.9–10.4	9.1–10.3	***5.2–7.4***	10.9	9.9–11.0	***6.2–7.6***
Genotype4d	7.9–10.9	***5.7–7.1***	7.9–10.3	10.4–10.9	***6.2–6.7***	10.4–11.0
Genotype4e	8.1–11.5	9.1–10.3	9.3–11.7	10.6	10.1	10.9
Genotype4f	8.7–12.5	11.5–12.7	8.9–10.3	10.5–12.9	10.2–11.0	8.1–8.9
Genotype4g	9.7–12.1	9.5–11.7	9.2–11.7	10.1	9.6	9.8

The minimal value of each column was listed in bold italics.

**Figure 2:**
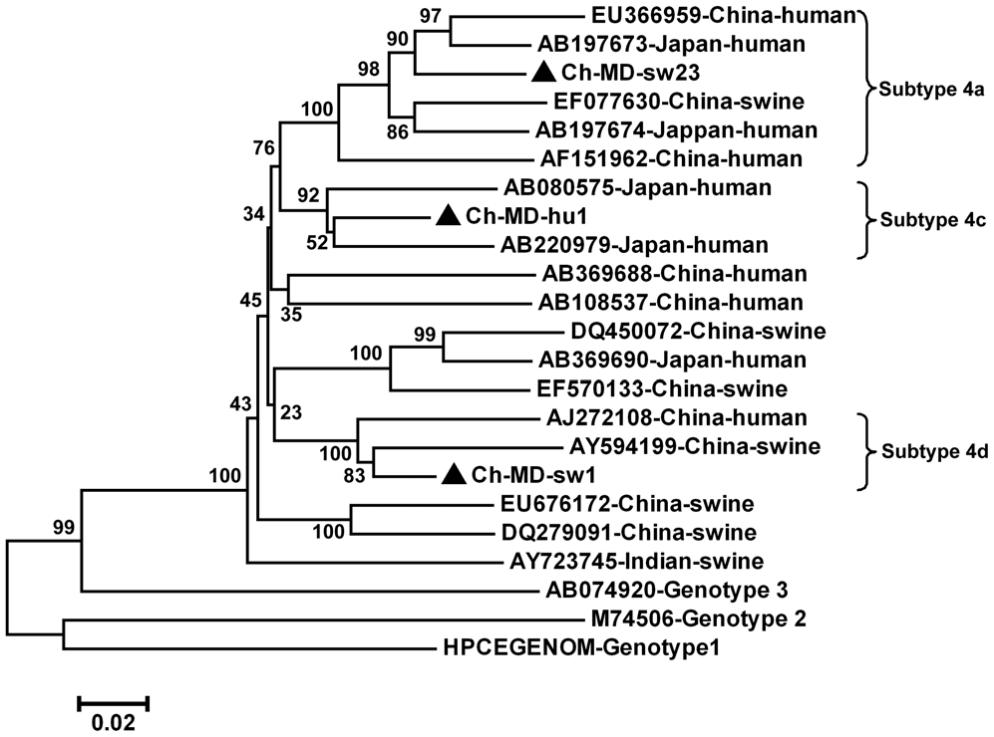
The phylogenetic tree constructed by alignment of the complete 2025-nt nucleotide sequence of ORF2 using Mega 4 software. Virus strains included the three isolates in this study and the referenced genotype 4 isolates. Percent bootstrap support is indicated at each node. GenBank accession no., source and country of origin are indicated. Genotype 1, 2 and 3 strains are included as outgroup. The isolates identified in this study were marked with black triangles.

From the three major groups, three representative strains, Ch-MD-sw1, Ch-MD-sw23 and Ch-MD-hu1, were selected and their complete ORF2 sequences were determined. Sequence analysis based on the complete ORF2 sequence indicated that the two swine HEV strains, Ch-MD-sw23 and Ch-MD-sw1, shared 91.7% sequence homology with each other, and 89.7% and 89.3% with the human isolate Ch-MD-hu1, respectively. Their putative amino acid sequences shared 98.5–99.4% sequence identity. Phylogenetic analysis was performed based on the three representative isolates in the present study and 17 referenced, and well characterized, genotype 4 HEV isolates [16]. The Ch-MD-hu1 strain clustered together with 2 Japanese human genotype 4 isolates (AB080575 and AB220979) with a 92% bootstrap value and shared 93.0% and 92.2% sequence identity with them, respectively, which classified Ch-MD-hu1 as genotype4c. The swine isolate Ch-MD-sw1 clustered together with two Chinese isolates (AJ272108 and AY594199) with a 100% bootstrap value and they shared a 90.8% and 92.4% sequence identity, respectively, which indicted Ch-MD-sw1 belonged to genotype4d. The other swine HEV isolate, Ch-MD-sw23, clustered with 5 other Chinese or Japanese genotype 4 strains with a 100% bootstrap value and they shared 92.3-93.6% sequence identity. The references used here defined Ch-MD-sw23 as genotype4a. The three columns on the right of table 4 show the genetic differences between the three strains whose entire ORF2 was sequenced in the present study and those of all the full genome strains available in GenBank calculated over the full ORF2 region. We conclude that Ch-MD-sw1, Ch-MD-sw23 and Ch-MD-hu1 show the closest sequence identity with genotype4d, genotype4a and genotype4c, respectively, which is consistent with the results of the phylogenetic analysis based on the entire ORF2 region (Fig. 2).

Combining these results, it suggests that there are two different subgenotypes of genotype 4 HEV strains prevalent in swine populations in central China, and they are genetically distinct from the strains which are prevalent in general human populations in this area.

### Dot-Blot Assay

**Figure 3:**
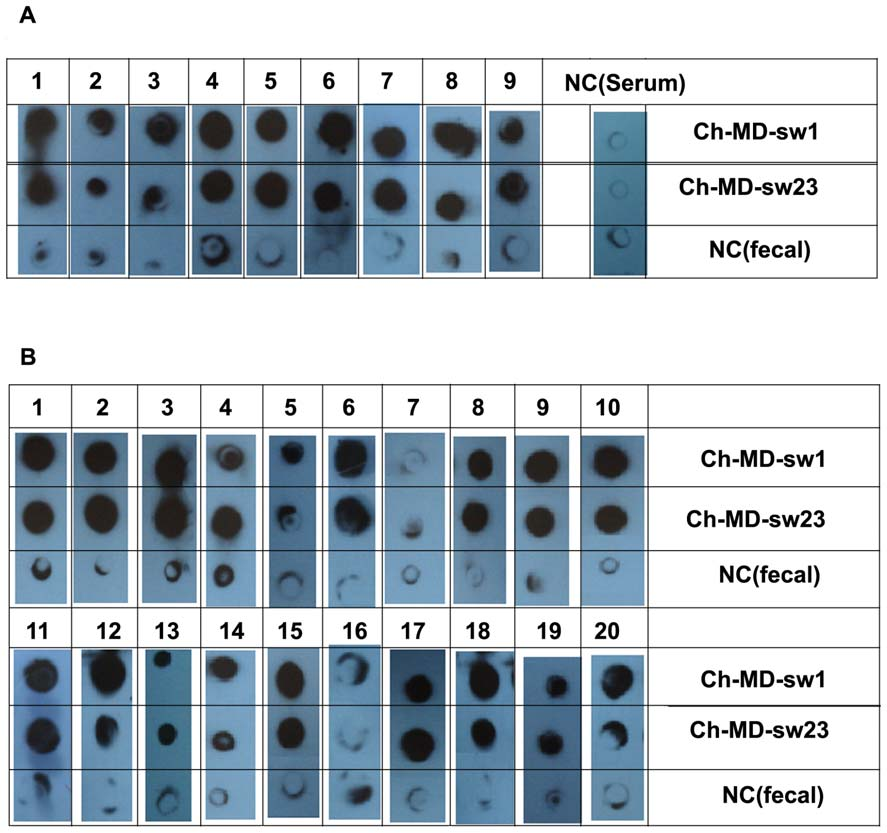
The results of Dot-blot assay. (A) The Dot-blot assay using the 9 serum samples which were positive for IgM, IgG and HEV RNA; (B) The Dot-blot assay using the 20 randomly selected serum samples which were positive for anti-HEV IgG but not for IgM and HEV RNA. Line NC(fecal) mean presents the reactivity between HEV-negative fecal suspension and the serum samples; Column NC(serum) presents the reactivity between HEV antibody negative serum sample with the fecal suspension.

The reactivity of the two representative swine HEV strains (Ch-MD-sw1 and Ch-MD-sw23) with the 29 serum samples which were positive for anti-HEV IgG was determined by Dot-blot assay. Fig. 3A presents the Dot-blot assay using the 9 serum samples which were positive for IgM, IgG and HEV RNA. Results show a strong reaction between the 9 sera and the two representative HEV strains in the dot-blot assay. Fig. 3B presents the Dot-blot assay using the 20 randomly selected serum samples which were positive for anti-HEV IgG but not for IgM or HEV RNA. Results showed that 19 (95%) of them reacted with the two HEV strains. None of the negative controls showed evident reactivity in the assay.

## Discussion

There is gathering evidence that HEV is enzootic and pigs are considered one of the major reservoirs of human infection with this virus. The zoonotic transmission of HEV from pigs to humans has been suggested previously, particularly for cases in non-endemic areas. This hypothesis was mainly based on phylogenetic analysis, which showed that swine and human HEV strains from same geographic regions share a high genomic similarity [9], [17]–[19]. Tei et al. provided direct evidence of zoonotic transmission of HEV from deer to human: patients became infected with HEV after eating raw meat of an infected deer [20]. A similar scenario has also been documented [21]: a patient was hospitalized after eating wild boar meat. Furthermore, there was only one nt difference between the sequence from the patient and the wild boar meat over the 1,980 nt of the entire ORF2 giving a nucleotide sequence identity of 99.95%.

Based on the phylogenetic analysis of the 150 nt partial ORF2 HEV sequences from both humans and pigs, Zheng et al reported that genotype 4 HEV transmitted freely between swine and humans in eastern China [9]. In the present study, our research was conducted in three swine farming districts in the central China, however, the HEV strains prevalent in swine (Fig. 1, group1 and 2) were genetically distinct from those prevalent in the human populations (Fig. 1, group3). This was confirmed by the phylogenetic analysis of the complete ORF2 genome sequence of three representative strains from the three groups, though these 53 strains all belonged to genotype 4 virus. Previous analysis has shown the 150-nt ORF2 sequence used in this study to be phylogenetically informative and to give similar trees to those based on the entire viral genome [22], [23], and on this we base the phylogenetic relationship of the 44 isolates in the present study established in the radial phylogenetic tree (Fig. 1).

From the sequencing results, some of the 39 swine HEV strains showed non-unique sequences over the 150nt region. Among these were four strains which had identical sequences but came from two different farms. Phylogenetic analysis also indicated that some of swine or human strains were very closely related (e.g. Ch-MD-sw4, 7 and 10 in group 1, and Ch-MD-hu4, 6 and 11 in group3 in Fig. 1), although our sampling/questionnaire records showed that not all of the non-unique or closely related strains were collected from the same farm or households. These probably represent an outbreak with the same HEV strain within this area or, imported via other transmission routes.

Hepatitis E is classically a water-borne infectious disease. Water contaminated by hepatitis E virus (HEV) that is being shed enterically from humans and animals may lead to large outbreaks of Hepatitis E [13], [24]. Recent studies suggested that genotype 4 virus freely circulated among swine and humans in eastern China where there are many rivers. One study found that persons living in communities downstream of swine farms were at 29% higher risk of infection than persons living in communities upstream [9]. Therefore, water contaminated by HEV may contribute to the zoonotic transmission from swine to human in eastern China.

Over the last few years there have been a flurry of case reports, case series and descriptions of small scale outbreaks relating to hepatitis E from Japan [20], [21]. There, HEV is enzootic among deer, boars and swine, and disease is linked to the eating of raw venison and boar liver, and of inadequately cooked boar meat and pig liver. The evidence appears to indicate that hepatitis E in Japan is a food-, not water-, borne disease, and also a zoonosis.

In the present study, our research was conducted in central China, which is an area of plains with few rivers. Most of the local rural residents consume deeply drilled borehole water. Moreover, the residents in this area are not known to have a penchant for eating raw meat. All these factors make the circulation of HEV in swine or humans independent in central China and thus significantly reduce the chance of HEV cross-species transmission from animals to human. Whether there are other factors which hinder human HEV strains from infecting pigs or vice versa will need to be elucidated by further research.

In the present study, all the human serum samples were collected from the general population. None of the 24 persons who were positive for anti-HEV IgM showed evident clinical symptoms of hepatitis, and only two of the 98 persons who were only positive for anti-HEV IgG had a history of being diagnosed with hepatitis E. This suggests that HEV infection in central China is commonly sub-clinical. Thus, HEV infection may persist in disease-endemic populations by serial transmission among susceptible persons in the form of subclinical and asymptomatic infection. These subclinical cases, which go unnoticed, may act as one of the reservoirs for persistence of this infection in the population and as a source of sporadic acute hepatitis E. A similar continual transmission of subclinical infection was the main reservoir of poliovirus in areas in which the disease was endemic. Subclinical HEV infection in laboratory infected cynomolgus macaques was observed by Aggarwal, who reported that subclinical HEV infection in some animals was associated with failure of the development of an immune response when compared with animals with clinical HEV infection [25]. Further study should be performed to elucidate whether the phenomenon occurs routinely during human subclinical HEV infection.

Most of the evidence supporting animal–human transmission of HEV is from regions where HEV infection is not endemic. In India, an endemic area for HEV, some reports indicate that zoonotic transmission of HEV from pigs to humans does not exist, and the strains prevalent in human populations all belong to genotype 1 HEV while those prevalent in pigs belong to genotype 4 [26], [27]. The genotype 1 HEV strains were proven to be unable to to infect pigs [28], [29]. Therefore, the phenotypically distinct genotypes could be used to interpret why zoonotic transmission was not observed in India.

In the present study, although HEV strains prevalent in human and swine groups in central China all belonged to genotype 4, and the complete ORF2 sequence of representative strains shared nucleotide and amino acid identity of more than 89% and 98%, respectively, they clustered separately into genetically distinct clusters. This suggested that the swine and human HEV isolates in central China are distinct from each other, this phenomenon is not attributed to virus genotype but non-viral factors, such as human living habits, living environments, and local climate.

Phylogenetic analysis based on the full or partial ORF2 sequences showed that the human or swine isolates in the present study cross clustered with the swine and human isolates from other regions of China and even Japan (Fig. 1 and 2), which may be due to travel of infected persons or or trading of infected pigs between China and Japan. This phenomenon should be elucidated by future research.

So far, genotype 1 and 4 HEV viruses have been found to be prevalent in human populations in China where genotype 4 HEV has become the dominant cause of hepatitis E disease since 2000 [14]. Zheng et al. isolated 21 HEV strains in the human sera collected from 2002 to 2005 in eastern China [9]. Only 4 of the 21 HEV strains belonged to genotype 1, and all 4 strains were isolated from the sera collected in 2002.

In the present study, we used the primers which had been optimized for genotypes 1 and 4 virus RNA detection [22], but all the 14 HEV strains that were isolated from the sera collected from central China belonged to genotype 4, suggesting genotype 4 HEV is the main genotype prevalent in this area. Although the primers also afford sensitive detection for genotype 3 strains, a genotype that was recently reported to be prevalent in swine groups in some areas of China [19], [20], we did not detected genotype 3 strains among the 554 swine fecal samples collected from central China.

In the present study, the age group 20–39 years had the highest positive rate of IgM, suggesting that people in this age group have the highest clinical attack rates in central China. This is consistent with the current opinion that the peak incidence in sporadic cases of hepatitis E in endemic regions occurs in 15-35-year-olds [31], [32]. IgG seropositivity reaches a plateau in the age group 40–59 years (Table 2) which was consistent with a recent age-prevalence study performed in southern China that indicated that the highest seropositivity was recorded in the age group 45–55 years [15]. A similar conclusion was reached in Japan where seropositivity reaches a plateau in the age group 40–49 years [33]. Specific IgG is usually produced early in infection and concentrations rise rapidly afterwards [34]. Estimates of the duration of the IgG response and immunity to subsequent infection vary, but the antibody has been detected for at least 12 years after acute infection [35]. Therefore, we believe that the higher positivity rate of IgG in older people (>40 years) is mostly due to infection earlier in life (20–39 years).

HEV has been isolated and fully sequenced. Sequence analysis of the various genotypes of HEV isolates shows significant similarity of the genomes and many cross-reactive epitopes between these different genotypes. It has been shown that antibodies to one strain are cross-reactive with the other strains, indicating cross-protection between strains. The anti-HEV assays used in the current study used a recombinant peptide of a genotype 1 HEV structural protein that occurs naturally as a homodimer [36]. Previous studies have shown that the IgM antibody detected by the assay is a reliable marker of recent HEV infection and that the seroprevalence of IgG antibody reflects the cumulative exposure of a study population to HEV [37], [38]. In the current study, the reactivity of the two representative swine HEV with antibodies in their serum samples were tested by Dot-blot assay. The results suggested that the anti-HEV antibodies prevalent in human populations can cross-protect against the strains prevalent in swine groups in central China.

In conclusion, the present study investigated the HEV infection status in the general human population and swine groups in central China. The positive rate of anti-HEV IgG and IgM of the studied subjects was 7.9% (117/1476) and 1.6% (24/1476), respectively. HEV RNA was detected in 0.95% (14/1476) of the general human subjects and 7.0% (39/554) of the swine stool samples. Phylogenetic analysis indicated that although all the HEV strains prevalent in central China belonged to genotype 4, the strains prevalent in swine were genetically distinct from those prevalent in the human populations, which suggested that cross-species transmission of HEV between human and swine might not exist in this area.

## Materials and Methods

### Sampling

From September in 2007 to September in 2008, a total of 1476 serum specimens were collected from general populations (779 males and 697 females, age range 2-79 years) of rural communities in central China, including Fuyang ( lies on the boundary of Anhui and Henan Province), Huaibei (lies on the boundary of Anhui and Shandong Province ), and Suzhou (lies on the boundary of Anhui and Jiangsu Province). Most of the participants in the present study were farmers who mainly working on crops growing, and agricultural products processing. All participants ≥16 years of age gave written consent after receiving a full explanation of the study, and written consent was also obtained from the parents of the participants <16 years of age. The Ethics Committee of Shanghai Research Center for Biomodel Organism approved the study. A questionnaire was used to record individual's history of ever being admitted to a hospital because of hepatitis E and frequently tripping to other regions. A total of 554 fecal samples were obtained from pigs aged from 4 to 26 weeks old from March to August in 2008. The swine fecal samples were obtained from 11 swine farms in Fuyang, Huaibei, and Suzhou. Fuyang lies about 200 km from Suzhou and Huaibei. Huaibei lies about 100 km from Suzhou. All the swine farms are formalized and have good sanitation status.

### Detection of Anti-HEV IgG and IgM

Anti-HEV IgG and IgM levels were determined using commercial ELISAs (Wan Tai Pharmaceutical, Beijing, China). The ELISAs were performed with a recombinant peptide corresponding to aa 394–606 of the major structural protein specified by open reading frame (ORF) 2 of the HEV genome. Testing was performed in accordance with the manufacturer's instructions.

### Detection of HEV-RNA

Total RNA was extracted from 100 uL of serum sample or feacal suspension, using Trizol (Invitrogen, USA). RT-PCR was performed as described elsewhere [22]. Briefly, a 150-nt segment of ORF2, nt 6317–6466 relative to HEV D10330, was amplified using primers E1 (5′-CTGTTTAAYCTTGCTGACAC- 3′) and E5 (5′-WGARAGCCAAAGCACATC-3′) for the first round of PCR and primers E2 (5′-GACAGAATTGATTTCGTCG- 3′) and E4 (5′-TGYTGGTTRTCRTAATCCTG-3′) for the second round. PCR cycling conditions for both rounds consisted of 35 cycles of denaturation for 30 s at 94°C, annealing for 30 s at 53°C, and extension for 40 s at 72°C.

### Determination of the Full Nucleotide Sequences of ORF2

The complete sequences of ORF2 of the three representative strains of the three major clusters in the phylogenetic analysis were determined using RT-PCR method with the primers designed according to the related sequences available in GenBank.

### Sequencing

Specific bands for HEV were purified from 1% agarose gel using the QIAquick Gel Extraction kit (Qiagen, Gemany). The purified PCR products were ligated into pMD18-T Vector (TaKaRa, Japan). For each product, three positive colonies were selected and sequenced. A consensus sequence of each fragment was saved and used for phylogenetic analysis or assemble of the full ORF2 sequence.

### Sequence and Phylogenetic Analysis

The viral nucleotide or putative amino-acid sequences were aligned using ClustaX1.8 or DNAstar software. Phylogenetic trees were constructed using the neighbor-joining method and evaluated using the interior branch test method with MEGA software (version 4.0; available at: http: //www.megasoftware.net/). Prototype HEV strains used as references in the analysis and their GenBank accession numbers and source of origin are marked in the phylogenetic tree.

### Dot-Blot Assay

To test the reactivity between the anti-HEV IgG detected in human sera and HEV particles in the swine fecal suspensions. The 9 serum samples which showed positive for both anti-HEV IgG and HEV RNA, and 20 of the 98 serum samples which showed positive only for anti-HEV IgG were selected and examined by Dot-blot assay using two representative strains: Ch-MD-sw1 and Ch-MD-sw23, whose complete ORF2 sequences were determined in the present study. A HEV-negative swine fecal suspension was included as a negative control. Briefly, HEV-positive swine feacal specimens were converted to 10% (w/v) suspensions in PBS, and clarified by centrifugation at 10000 g for 10 min. Then about 30 ul suspension were spotted onto PVDF membrane strips. Blots were blocked by shaking the at 37°C for one hour with TBS-T (2% Tris–HCl pH 7.5, 140 mM NaCl, 0.1% Tween 20) containing 5% skim milk powder. Then, blots were washed three times with TBS-T and incubated with each serum diluted 1∶200 in TBS-T (1 h at room temperature with constant shaking). A human serum negative for anti-HEV IgG were included as negative control. After incubation, blots were washed four times with TBS-T, and incubated with a horseradish peroxidaseconjugated goat anti-human IgG (Dingguo, Beijing, China) at a 1∶10,000 dilution. After 1 h of incubation at room temperature, blots were washed four times with TBS-T and color development was carried out using a chemiluminescence detection reagent (ECL AdvanceTM Western Blotting Detection kit, GE Healthcare Bio-Sciences).
